# Efficacy, Kinetics, and Mechanism of Tetracycline Degradation in Water by O_3_/PMS/FeMoBC Process

**DOI:** 10.3390/nano15141108

**Published:** 2025-07-17

**Authors:** Xuemei Li, Qingpo Li, Xinglin Chen, Bojiao Yan, Shengnan Li, Huan Deng, Hai Lu

**Affiliations:** 1 College of Visual Arts, Changchun Sci-Tech University, Changchun 130600, China; 100464@cstu.edu.cn (X.L.); 100404@cstu.edu.cn (B.Y.); lishengnan@neau.edu.cn (S.L.); 100343@cstu.edu.cn (H.D.); 2 Jinjiang Industrial Group, Jinjiang 362261, China; liqingpo@student.jlju.edu.cn; 3 Key Laboratory of Songliao Aquatic Environment, Ministry of Education, Jilin Jianzhu University, Changchun 130118, China; chenxinglin@student.jlju.edu.cn

**Keywords:** tetracycline degradation, O_3_/PMS/FeMoBC, efficacy, kinetics, mechanism

## Abstract

This study investigated the degradation efficacy, kinetics, and mechanism of the ozone (O_3_) process and two enhanced O_3_ processes (O_3_/peroxymonosulfate (O_3_/PMS) and O_3_/peroxymonosulfate/iron molybdates/biochar composite (O_3_/PMS/FeMoBC)), especially the O_3_/PMS/FeMoBC process, for the degradation of tetracycline (TC) in water. An FeMoBC sample was synthesized by the impregnation–pyrolysis method. The XRD results showed that the material loaded on BC was an iron molybdates composite, in which Fe_2_Mo_3_O_8_ and FeMoO_4_ accounted for 26.3% and 73.7% of the composite, respectively. The experiments showed that, for the O_3_/PMS/FeMoBC process, the optimum conditions were obtained at pH 6.8 ± 0.1, an initial concentration of TC of 0.03 mM, an FeMoBC dosage set at 200 mg/L, a gaseous O_3_ concentration set at 3.6 mg/L, and a PMS concentration set at 30 μM. Under these reaction conditions, the degradation rate of TC in 8 min and 14 min reached 94.3% and 98.6%, respectively, and the TC could be reduced below the detection limit (10 μg/L) after 20 min of reaction. After recycling for five times, the degradation rate of TC could still reach about 40%. The introduction of FeMoBC into the O_3_/PMS system significantly improved the TC degradation efficacy and resistance to inorganic anion interference. Meanwhile, it enhanced the generation of hydroxyl radicals (^•^OH) and sulfate radicals (SO_4_^•−^), thus improving the oxidizing efficiency of TC in water. Material characterization analysis showed that FeMoBC has a well-developed porous structure and abundant active sites, which is beneficial for the degradation of pollutants. The reaction mechanism of the O_3_/PMS/FeMoBC system was speculated by the EPR technique and quenching experiments. The results showed that FeMoBC efficiently catalyzed the O_3_/PMS process to generate a variety of reactive oxygen species, leading to the efficient degradation of TC. There are four active oxidants in O_3_/PMS/FeMoBC system, namely ^•^OH, SO_4_^•−^, ^1^O_2_, and •O_2_^−^. The order of their contribution importance was ^•^OH, ^1^O_2_, SO_4_^•−^, and •O_2_^−^. This study provides an effective technological pathway for the removal of refractory organic matter in the aquatic environment.

## 1. Introduction

With the development of science and technology and industry, antibiotics are produced in large quantities and widely used, which leads to the detection of antibiotics and their transformants in the water environment of more and more countries and regions [1]. Antibiotics have a low absorption rate in human and animal bodies and are eventually excreted in the form of feces and urine. Antibiotics and their conversion products (conjugated state, oxidation products, hydrolysis products, etc.) are released into surface water and groundwater. Other sources of antibiotics include wastewater discharged from hospitals and pharmaceutical factories. TC is a typical kind of antibiotic. TC and its salts have good hydrophilicity and biological stability, so it is easy to migrate between different environmental media and eventually remain in the environment for a long time, which will pollute the environment to a great extent. According to the survey of antibiotic pollution in dozens of countries around the world, water has become one of the most important enrichment places of antibiotics in the environment. TC is widely used in the world because of its low price. Its types mainly include chlortetracycline, oxytetracycline, tetracycline, and semi–synthetic derivatives such as methotrexate, doxycycline, and minocycline [2]. The solid–liquid partition coefficient (K_d_ value) of TC antibiotics is 4.2 × 10^4^~1.03 × 10^5^ mg/kg, which is higher than other antibiotics. Therefore, TC antibiotics that enter the environment are more likely to be adsorbed and accumulated in the soil, thus destroying the microbial community in the soil environment, inhibiting the growth of beneficial microorganisms in the soil, and accelerating the spread of resistance genes. This may lead to the large-scale spread of resistance genes in the water environment. Therefore, it is urgent to develop technologies to remove antibiotics from the environment [1,2,3].

The ozone (O_3_) process has been widely used in the water treatment industry and is commonly used for the removal of organics, odors, and coloration from water [4,5,6]. Although the traditional O_3_ process has high degradation efficiency, it also has some defects, such as high energy consumption, a low effective conversion rate of ozone (saturated solubility), poor mineralization ability, and a poor anti-interference ability for inorganic ions, resulting in a large number of intermediate products in the secondary effluent of sewage treatment plants [6]. An effective strengthening process for the O_3_ process is the O_3/_peroxymonosulfate (O_3_/PMS) process [7]. In this process, O_3_ acts as an oxide to activate PMS, so it can produce •OH and SO_4_^•−^ at the same time, showing the advantages of rapidly degrading organic pollutants and mineralizing them into CO_2_ and H_2_O, which has great advantages compared with the traditional advanced oxidation process. SO_4_^•−^ in the O_3_/PMS process is mainly provided by PMS. SO_4_^•−^ has a high redox potential, which is equivalent to •OH (2.5–3.1 eV) under neutral conditions but is slightly higher than •OH (1.9 eV) under acidic conditions. At the same time, it has a long half-life (30–40 μs) and a wide pH application range. Under a normal environment, the decomposition rate of persulfate anions (S_2_O_8_^2−^) to produce SO_4_^•−^ is slow, and the effect of using PMS alone is not good. Therefore, many methods of activating PMS were derived, including thermal decomposition, ultrasonic wave, ultraviolet radiation, alkali activation, laser flash activation, and transition metal catalysis [8].

In order to further enhance the coupling of O_3_ with PMS and to solve the problems of low O_3_ utilization, low mineralization, bromate generation, and low resistance to inorganic ions in the O_3_/PMS process, an iron molybdates/biochar composite (FeMoBC) was prepared to modify the O_3_/PMS process in this study. Therefore, the zone/peroxymonosulfate/iron molybdates/biochar composite (O_3_/PMS/FeMoBC) process was introduced. Studies have shown that the iron molybdates composite is environmentally friendly because it contains nutrients such as iron and molybdenum which are necessary for human body and plant growth, and the existence of them can promote redox and make it form multivalent states (Fe valence ranges from 0 to +3, and Mo valence ranges from 0 to +6) [9]. In addition, metal molybdate has attracted wide attention because of its high stability, high electrochemical activity, and special structure, and ferrous molybdate (FeMoO_4_) is one of the representative materials [10]. Meanwhile, iron and molybdenum have good activation efficiency for O_3_ and PMS [11]. The existence of bimetals increased the electron transfer rate in the reaction system and further improved the catalytic reaction rate of the system. In order to control the ion leaching of bimetallic substrate in water and keep the metal ions in a low valence state after calcination, BC with a low cost and less energy consumption was used as its carrier. Using typical agricultural wastes (corn stalk) in Northeast China and Fe/Mo mixed salt as raw materials, metal BC composites were prepared by wet impregnation pyrolysis. In this way, bimetallic oxides were loaded on the pores and surfaces of BC, which reduced the leaching of metal ions. Meanwhile, carbon materials can also inhibit the production of bromate in the O_3_/PMS process. In this process, iron ions and molybdenum ions have good activation efficiency for O_3_ and PMS, and the existence of bimetal increases the electron transfer rate in the reaction system, which further improves the catalytic reaction rate of the system.

Furthermore, the efficacies of these three process technologies, O_3_, O_3_/PMS, and O_3_/PMS/FeMoBC, for the degradation of TC in water were studied, respectively, by investigating the initial concentration of TC, the O_3_ concentration, the pH, the initial concentration of PMS, the amount of material dosage, and four common inorganic anions in water (HCO_3_^−^, NO_3_^−^, Cl^−^, and SO_4_^2−^) and humic acid (HA) on the reaction. Meanwhile, the reaction kinetics were calculated and fitted to the specific degradation effect of each single-factor experiment, and the degradation efficacy of the three systems was analyzed by their degradation efficiencies and chemical reaction kinetic rates within 20 min, and then the mechanism of the oxidative degradation of TC in water by the O_3_/PMS/FeMoBC process was explored. Through this study, we aimed to put forward a strengthening measure of the O_3_/PMS process to improve its degradation efficiency for TC. Meanwhile, it also can provide a new idea for the removal of refractory organic matter in water and has important practical significance for improving the quality of the water environment.

## 2. Materials and Methods

### 2.1. Materials and Reagents

The main reagents used in this experiment are detailed in Table 1.

All solutions in the experiments were prepared by an ultrapure water machine (Molelement elemental type 1820a water system, Molecular scientific instrument limited company, Shanghai, China). Corn stalks were purchased from a farm in Changchun, China. They were washed and dried, crushed into powder by a crusher, and screened by a 100-mesh sieve. Then they were put into a vacuum oven for pyrolysis at 200 °C for 120 min and then packaged in self-sealing bags for later use. The iron and molybdenum sources were iron chloride (FeCl_3_•6H_2_O) and sodium molybdate (Na_2_MoO_4_•2H_2_O), respectively. MoBC, FeBC, and FeMoBC samples were synthesized by the impregnation-pyrolysis method. The preparation process of MoBC was as follows: a suitable amount of BC was immersed into 500 mL solution containing Na_2_MoO_4_•2H_2_O solutions with different molar mass ratios (C:Mo = 1:1, 1:1.5, 1:2). The mixture was placed in an 80 °C constant temperature water bath and stirred for 12 h. After cooling, they were centrifuged at 4000× *g*, separated, and then dried in a 70 °C vacuum drying oven. The materials were then ground into powder. Then the samples were calcined in a muffle furnace at different temperatures (500, 600, and 700 °C) for 60/120 min and then cooled to room temperature and taken out, thus obtaining the MoBC samples. The above samples were crushed, ground, washed with water three times, and finally dried in vacuum at 70 °C. The ground and sieved samples were stored in brown bottles in a well-ventilated, cool place for later use. The preparation processes of FeBC and FeMoBC were similar to those of the above-mentioned MoBC samples, in which C:Fe = 1:1, 1:1.5, 1:2; C: Fe:Mo = 1:1:1, 1: 1.5, 1:1:2. The other preparation conditions remained the same.

### 2.2. Experimental Flow

**Figure 1:**
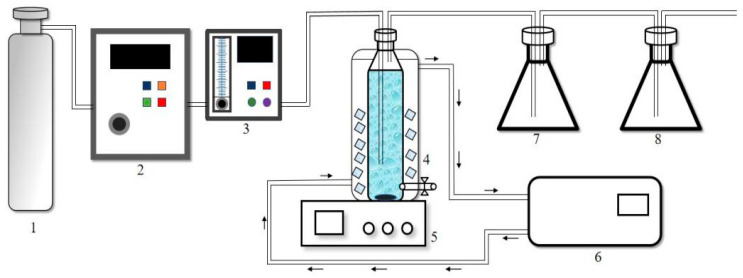
Reaction device diagram. (1)—O_3_ bottle; (2)—Ozonalor; (3)—rotameter, (4)—O_3_ water tank; (5)—magnetic stirrer; (6)—constant temperature system; (7)—Kl tail gas absorber; (8)—KI tail gas absorber.

This experimental setup is shown in Figure 1. The overall device consists of an O_3_ generator, a rotor flow meter, an air-drying dish, a magnetic stirrer with a constant temperature water bath, a sampler, and an exhaust gas treatment device. Wherein, a rotor was placed at the bottom of the O_3_ water tank for stirring (200 r/min). The volume of reaction solution was 1 L. The experimental temperature was controlled at 25 ± 1 °C. When sampling, the rubber stopper was removed, a certain amount of sample was sucked out with a syringe, and then the stopper was covered to avoid O_3_ leakage. In the tail gas absorption device, two brown gas cylinders were, respectively, filled with 500 mL of 20% potassium iodide solution, which was mainly used to collect excess O_3_ in preheating and reaction stages to avoid direct exposure to the environment.

### 2.3. Experimental Steps

(1) Before the start of the experiment, the pipeline was first connected to the tail gas treatment device, and the O_3_ generator was opened to the desired gear to run for 15 min to stabilize the O_3_ concentration in the subsequent reaction. At the same time, the water bath was preheated to 25 °C.
(2) Then the target reaction solution was added to the reactor, while magnetic stirring was turned on (with the aim of eliminating the dead water zone), and the pipeline valve was switched to the reactor.
(3) After the experiment started, samples were taken at 2 min intervals, 1 mL of water samples were taken each time, and 100 μL of Na_2_S_2_O_3_ standard solution (reaction terminator) was dropped into the sample bottle after sampling.
(4) The reaction solution was poured out 30 min after the end of the experiment to avoid the release of O_3_ from the water into the laboratory.

### 2.4. Analysis and Detection Methods

In this test, the iodometric method was used to determine the concentration of gaseous O_3_, following the iodometric method stipulated in Hygienic Requirements for Ozone Sterilizers (GB-28232-2020) [12]. TC detection was performed by high-performance liquid chromatography (HPLC, Agilent 1260 Infinity II, Santa Clara, CA, USA), with an ultraviolet detector and an Agilent zorbax SB-C18 column (4.6 × 150 mm, 5 μm), and the column temperature was controlled at 30 ± 1 °C. The mobile phase ratio was 0.1% formic acid water–methanol = 70:30 (*v*/*v*). The flow rate of mobile phase was 1 mL/min, the injection volume of samples was 50 μL, and the detection wavelength was 355 nm.

A TESCAN MIRA LMS scanning electron microscope (Quattro S, Brno, Czech Republic) was used to observe the morphology and energy spectrum of the samples. The analysis conditions were as follows: a small amount of samples were pasted on the conductive adhesive, and then metal spraying was carried out in a Quorum SC7620 sputtering coating instrument (Quorum SC7620, London, UK), with a gold spraying time of 45 s and a gold spraying current of 10 mA. The morphology and energy spectrum of the samples were observed by the TESCAN MIRA LMS scanning electron microscope mentioned above. The accelerating voltage for morphology observation was 3 kV, and the energy spectrum analysis was 15 kV. An SE2 secondary electron detector was used as the detector. The total specific surface area, total pore volume, and pore size data of the materials were determined using a Micromeritics ASAP2460 fully automated specific surface and porosity analyzer (ASAP2460, Norcross, GA, USA). During the determination, the nitrogen adsorption–desorption method was adopted. The degassing time was set to 8 h, and the temperature was kept at 200 °C. The functional groups on the surface of BC were tested using a Thermo Scientific Nicolet iS20 (iS20, Waltham, MA, USA). A proper amount of dried potassium bromide powder and 1/100~1/200 sample (relative to potassium bromide) were added into a mortar, fully ground, and pressed into transparent sheets by a tablet press. The test parameters were as follows: resolution was 4 cm^−1^; scanning time was 32 times, and test wavelength range was set to 400~4000 cm^−1^. A Rigaku SmartLab SE (SmartLab SE, Tokyo, Japan) was used to analyze the crystal structure before and after the reaction. A copper target was chosen as the test target. The scanning angle ranged from 5 to 90°, and the scanning speed was 5° min^−1^. XPS characterization was carried out using a Thermo Scientific K–Alpha XPS (K–Alpha, Waltham, MA, USA) instrument to differentiate the metallic valence states of Fe and Mo on the surface of the material. First, a proper amount of dried samples were taken out and pressed and then were put into the sample room after being stuck on the sample tray. When the pressure in the sample chamber was less than 2.0 × 10^−7^ mbar, the samples were sent into the analysis chamber. The spot size was 400 μm, the working voltage was 12 kV, and the filament current was 6 mA. In addition, the full-spectrum scanning pass energy was 150 eV, and the step size was set as 1 eV. The narrow-spectrum scanning pass energy was 50 eV, and the step size was 0.1 eV.

## 3. Structural Characterization of FeMoBC Material

### 3.1. SEM–EDS Characterization Analysis

**Figure 2:**
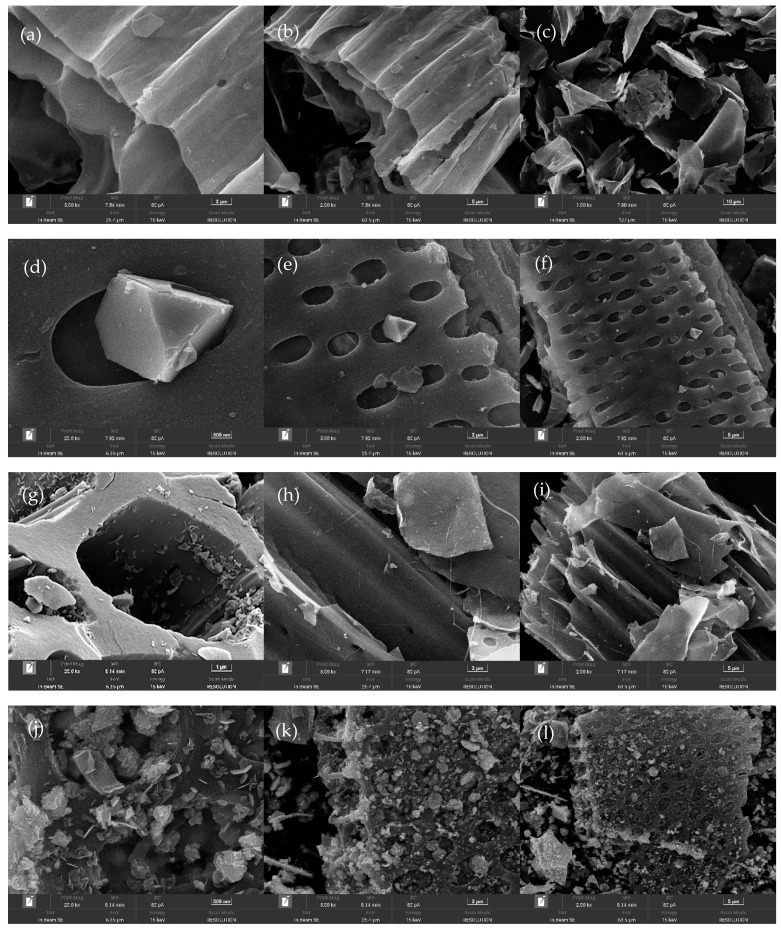

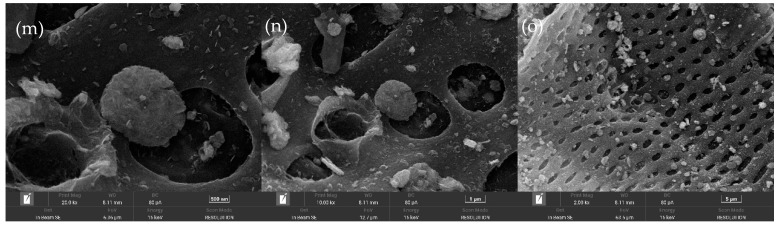
The SEM images at different multiples and scales. BC samples (**a**–**c**); FeBC sample (**d**–**f**); MoBC samples (**g**–**i**); FeMoBC (new) sample (**j**–**l**); FeMoBC (used) sample (**m**–**o**).

In order to compare the surface characteristics of the prepared BC, FeBC, MoBC, FeMoBC (unused), and FeMoBC (recycled three times), the structure and morphology of the five above-mentioned materials were analyzed by scanning electron microscopy. As shown in Figure 2, the pristine BC (Figure 2a–c) has a lamellar structure with a smooth outer surface, which is consistent with typical carbon structural features. Compared with the pristine BC, the surface of FeBC (Figure 2d–f) presents a porous, multilayered, and rough structure. However, MoBC (Figure 2g–i) only observed partial vanish of carbon structure pores and poor metal loading. However, the SEM image of FeMoBC (new) (Figure 2j–l) shows that the surface of the material was rough and irregular. Various metallic crystals were densely distributed on the surface, and metallic crystals were agglomerated in some places. At the same time, it can be clearly observed that irregular large particles were embedded in the pores, which was presumed to be metallic iron–molybdenum oxide particles produced during high-temperature calcination. Numerous metal particles were uniformly distributed on the surface and in the pores of the BC, and this phenomenon promoted the electron transfer of the catalyst in the oxidation experiments. In addition, compared with FeMoBC (new), the metal particles on the surface of FeMoBC (used) (Figure 2m–o) after the catalytic cycle of three degradation reactions were more uniformly distributed, which on the one hand indicates that a small part of the metal particles were dissolved during the reaction process, and on the other hand, it reflects that the Mo and Fe were stably loaded on the surface of the BC. This change in surface characteristics before and after the reaction confirmed that most of the reactions occurred on the catalyst surface.

**Figure 3:**
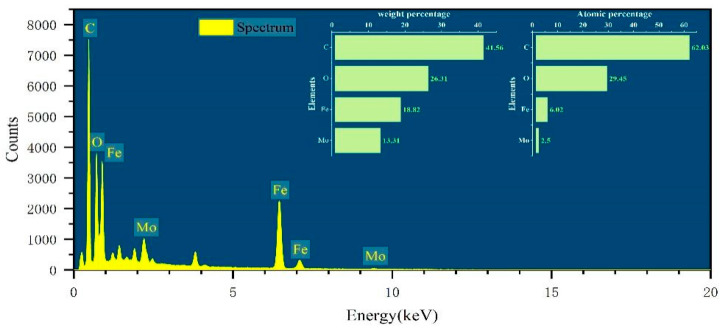
EDS spectra of FeMoBC.

The elemental composition of the FeMoBC material was clarified by EDS analysis. As shown in Figure 3, the elements of carbon, oxygen, iron, and molybdenum were present in the structure of FeMoBC, with mass percentages of 41.56%, 26.31%, 18.82%, and 13.31%, respectively. Meanwhile, their atomic weights were 62.03%, 29.45%, 6.02%, and 2.5%, respectively. This analysis confirmed that both metal Fe and Mo were evenly loaded on the surface and in the pores of the BC.

### 3.2. FTIR Characterization Analysis

**Figure 4:**
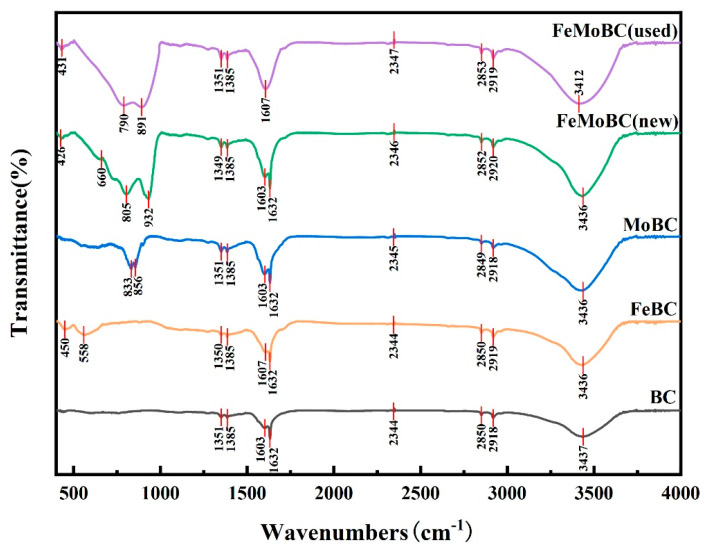
FTIR spectra of BC, FeBC, MoBC, FeMoBC (new), and FeMoBC (used) materials.

In order to investigate the surface functional groups of BC, FeBC, MoBC, FeMoBC (new), and FeMoBC (used), Fourier Transform Infrared Spectroscopy (FTIR) was applied to analyze them in the characteristic region of 400–4000 cm^−1^ (Figure 4). The results show that the relatively sharp peaks of BC, FeBC, MoBC, FeMoBC (new), and FeMoBC (used) at 3437, 3436, 3436, and 3412 cm^−1^, respectively, correspond to the vibration of the hydroxyl (–OH) linker. The vibration peaks around 2850 and 2920 cm^−1^, respectively, correspond to the bending vibration of alkanes (C-H) [13]. The new peaks in the FTIR spectra of FeBC, MoBC, and FeMoBC were generated, mainly due to the generation of new surface functional groups (Fe-O, Mo-O) after Fe/Mo co–loading. The C=C stretching vibration of the absorption peaks at 1603–1632 cm^−1^ is gradually enhanced by the modification of the BC material, while the strong absorption peak at 1351 cm^−1^ corresponds to the characteristic peak vibration of C-O [14]. It is noteworthy that two new absorption bands appear at about 450 cm^−1^ and 558 cm^−1^, which are usually considered as Fe-O, while the two new absorption bands at 805 and 932 cm^−1^ are Mo-O stretching vibration peaks [15]. Compared with FeMoBC (new), the intensity of the FeMoBC (used) part of the peaks is slightly attenuated, indicating that some of the functional groups are consumed in the catalytic reaction, and thus the catalytic reaction is mainly concentrated on the catalyst surface. The above phenomenon indicates that more Mo-and-Fe-containing oxygen functional groups were successfully loaded on FeMoBC.

### 3.3. BET Characterization Analysis

**Figure 5:**
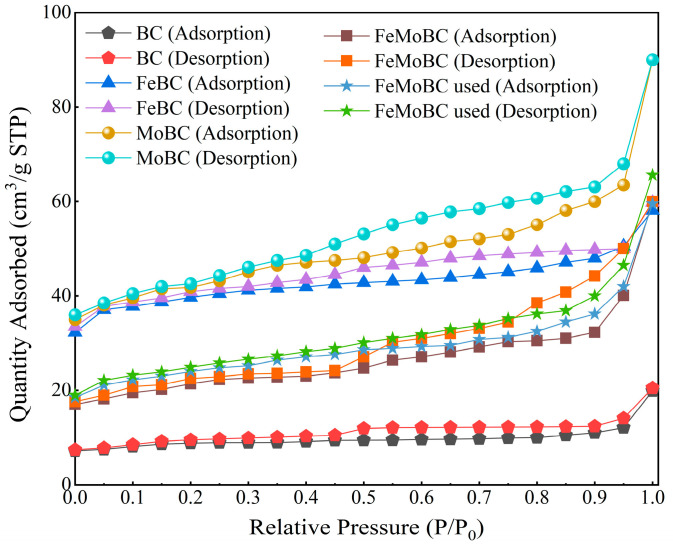
N_2_ adsorption–desorption isotherm diagram of BC, FeBC, MoBC, FeMoBC (new), and FeMoBC (used) materials.

The specific surface area of the catalyst reflects its porosity and general geometry, and the specific surface area, microporous area, and total pore volume of the catalysts are currently determined by N_2_ adsorption–desorption isotherm measurements. Figure 5 shows the N_2_ adsorption–desorption isotherm curves of BC, FeBC, MoBC, FeMoBC (new), and FeMoBC (used), which have a slowly increasing trend and belong to the typical type IV hysteresis loop isotherm. This reflects a partial microporous-to-mesoporous transition in the catalysts, indicating that the catalysts have more mesoporous structures with pore sizes predominantly between 2 and 50 nm. The pore properties of BC, FeBC, MoBC, FeMoBC (new), and FeMoBC (used) are shown in Table 2. The above results showed that the specific surface area, total pore volume, and average pore size of Fe/Mo metal-modified FeMoBC increased significantly compared with that of BC, but the specific surface area, total pore volume, and average pore size of FeMoBC were smaller than those of FeBC and MoBC, which may be attributed to localized agglomeration of some of the metal oxides on the surface of the BC and at the pore channels. This phenomenon is caused by the successful loading of Fe/Mo metal nanoparticles on the BC surface. Related studies have shown [16] that these aforementioned properties are sufficient to indicate that the coupling between the prepared composite FeMoBC and O_3_/PMS system has more positive effects, which are mainly reflected in the ability to enhance the electron transfer between the catalyst and the oxidant and to promote the degradation of pollutants into small molecules.

### 3.4. XRD Characterization Analysis

**Figure 6:**
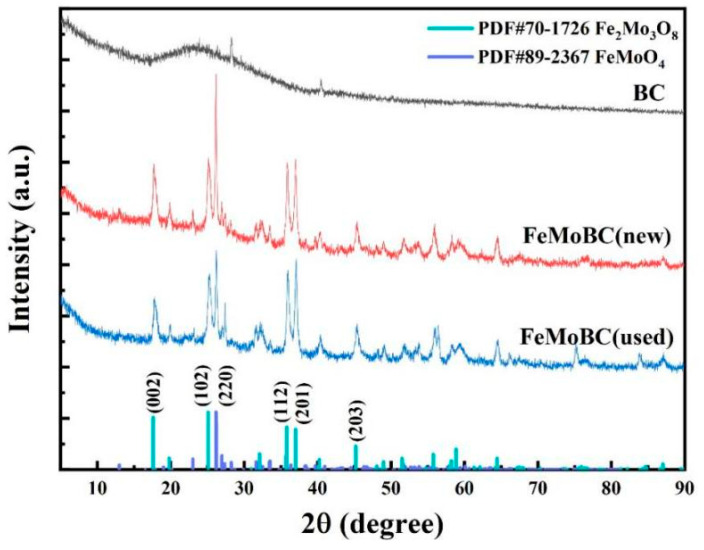
XRD spectra of BC, FeMoBC (new), and FeMoBC (used) materials.

The crystal structure of FeMoBC before and after use was determined by XRD patterns. The XRD patterns of BC, FeMoBC (new), and recycled-three-times-over FeMoBC (used) are shown in Figure 6. The XRD patterns show that BC has a typical planar graphite amorphous carbon structure (JCPDS 41-1487) with diffraction peaks located at 2θ = 24.5°, which usually has strong support properties for metal carriers. The FeMoBC diffraction peak rays are five, located at 2θ values of 17.62°, 25.08°, 35.82°, 37°, and 40.9°, and are highly compatible with (002), (102), (112), (201), and (203) of (Fe_2_Mo_3_O_8_) PDF#70–1726, respectively. The diffraction peak at 26.16° in the XRD diffraction pattern, along with weak peaks at 2θ values of 26.97°, 31.62°, and 33.52°, can be regarded as belonging to FeMoO_4_ according to the PDF standard card (PDF#89-2367). Compared with the pristine BC, the intensity of the diffraction peaks located at 23.94° was lower for FeMoBC (new) and FeMoBC (used), mainly due to the loading of metal ions that changed their surface morphology characteristics.

In addition, the diffraction peaks of the catalysts before and after the reaction were in the same position, but the peak intensities were slightly reduced, which indicated that the metal particles loaded on their surfaces were involved in the reaction. The analysis of the above two crystalline phases by jade 9.0 software yielded that the percentages of Fe_2_Mo_3_O_8_ and FeMoO_4_ were 26.3% and 73.7%, respectively. Therefore, the above XRD results indicate that the synthesis of FeMoBC catalyst was successful.

### 3.5. XPS Characterization

**Figure 7:**
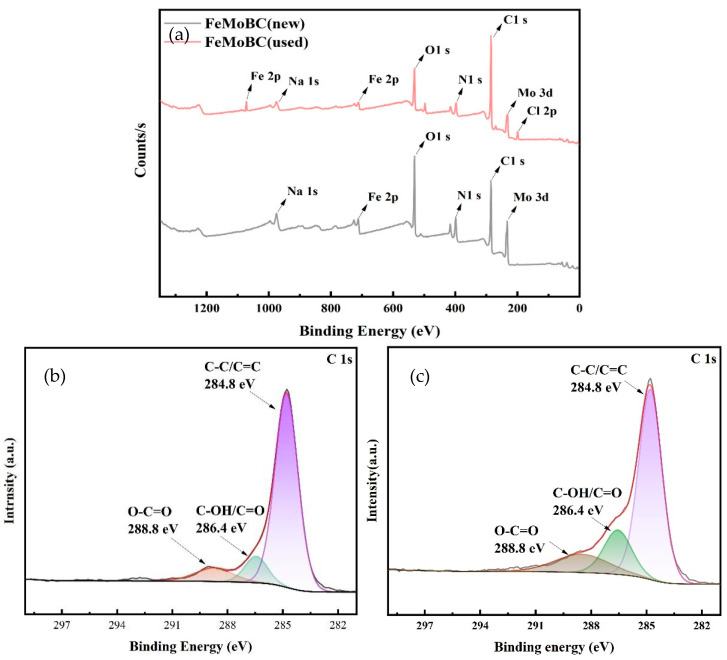

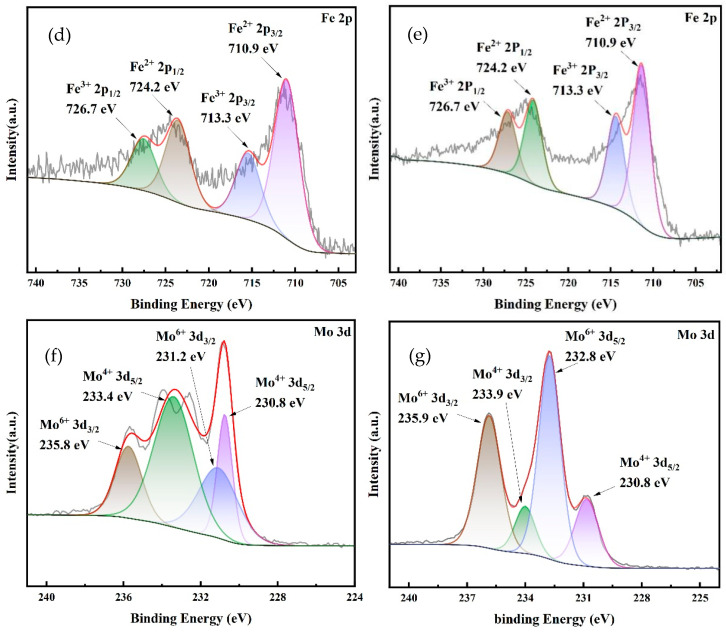
XPS of FeMoBC (new) and FeMoBC (used). (**a**) Full spectrum; (**b**) FeMoBC (new) C1s; (**c**) FeMoBC (used) C1s; (**d**) FeMoBC (new) Fe 2p; (**e**) FeMoBC (used) Fe 2p; (**f**) FeMoBC (new) Mo 3d; (**g**) FeMoBC (used) Mo 3d.

In order to further analyze the changes in the surface activity sites of the catalysts before and after the reaction, FeMoBC (new) and FeMoBC (used) were characterized by XPS spectroscopy, and the results are shown in Figure 7.

As shown in Figure 7a, in the full spectrum of the catalyst, the five peaks located at 285.08, 531.08, 712.08, 398.08, and 233.08 eV are higher, corresponding to C1 s, O1 s, Fe 2P, N1 s, and Mo 3d, respectively. Before and after the reaction, the intensity of peaks for the elements located in different orbitals of C, O, Fe, N, and Mo changed slightly, indicating that these substances were involved in the reaction. The C1 s, Fe 2P, N1 s, and Mo 3d plots show the changes in the surface activity sites and chemical composition of C, Fe, and Mo in FeMoBC catalysts before and after the reaction.

As shown in Figure 7b,c, the C1 s spectrogram has three types of characteristic peaks: a carbon–carbon double bond and carbon single bond (C=C/C-C, 284.8 eV), aldehydes and carbonyls (C-OH/C=O, 286.4 eV), and carboxyls (O-C=O, 288.8 eV). As shown in Figure 7d,e, there are two groups of characteristic peaks in the spectrum of Fe2p, namely Fe 2p_3/2_ and Fe 2p_1/2_. Among them, the satellite peaks of Fe 2p_3/2_ are 710.9 eV and 717.3 eV, and the satellite peaks of Fe 2p_1/2_ are 724.2 eV and 726.7 eV. Two peaks at 710.9 eV and 724.2 eV belonged to Fe (II), and 717.3 eV and 726.7 eV belong to Fe (III) [17,18,19]. The Fe (II, III) ion cycling is essential for the generation of ^•^OH in the Fenton-like system. The Fe2p spectra reflect that Fe (II, III) ion cycling occurs before and after the reaction. The peaks of the two sets of characteristic peaks in the Mo 3d spectra shown in Figure 7d,e correspond to Mo 3d_3/2_ and Mo 3d_5/2_. The Mo 3d_3/2_ and Mo 3d_5/2_ peaks at 230.8 and 233.4 eV, respectively, are attributed to Mo (IV), whereas the peaks at 231.2 and 235.88 eV are attributed to Mo (VI) and satellite peaks [20]. The peaks of Mo (IV) and Mo (VI) produce changes before and after the reaction, confirming the existence of a certain degree of electron transfer in Mo. Compared with FeMoBC (new), the peaks of FeMoBC (used) all had different degrees of attenuation after the cyclic reaction, suggesting that Fe and Mo were jointly involved in the reaction. The core of the decrease in Mo (IV) intensity was the reduction in the surface valence state induced by a reductive catalytic environment (such as Mo^5+^→Mo^4+^), supplemented by the influence of surface covering, ligand reconstruction, or bulk diffusion. It is also possible that surface passivation layer formation and competitive adsorption occurred. XPS characterization showed that multiple valence states of FeOx and MoOx were doped on the surface of the BC [21,22], which was in agreement with the results of the XRD detections and further illustrated the success of the FeMoBC synthesis.

### 3.6. Effect of Catalyst on TC Adsorption

**Figure 8:**
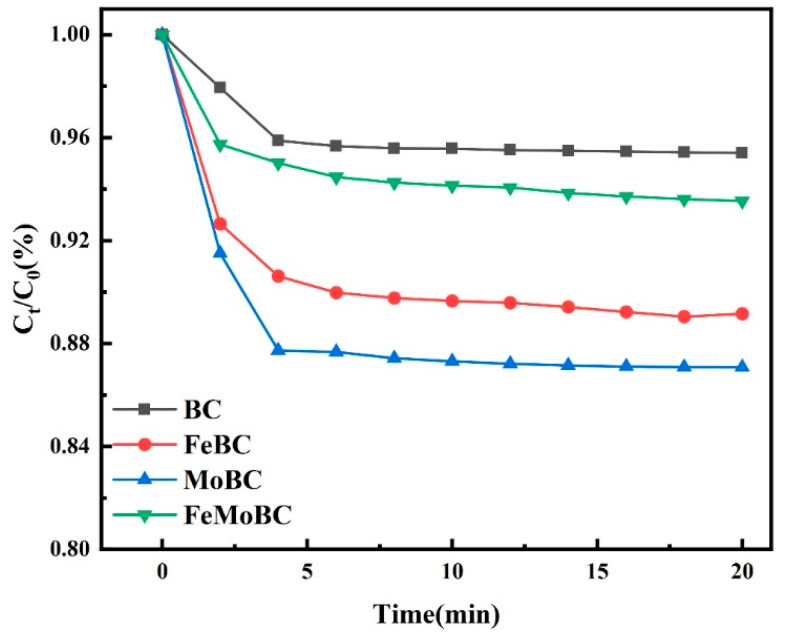
Adsorption properties of different materials for tetracycline.

Under the conditions of reaction temperature 25 ± 1 °C, material dosage 200 mg/L, rotor speed 200 r/min, initial TC concentration 0.03 mM, and solution pH 6.8 ± 0.1, the adsorption properties of BC, FeBC, MoBC, and FeMoBC for TC in water were investigated. As shown in Figure 8, the adsorption rates of TC by BC, FeBC, MoBC, and FeMoBC were 4.6%, 10.9%, 12.9%, and 6.47%, respectively, within 20 min. For FeMoBC, its adsorption capacity was lower than that of FeBC and MoBC, which might be due to the dense oxide crystals formed by bimetals in the pores of biochar materials. The previous characterization analysis also confirmed this result.

## 4. Efficacy of O_3_, O_3_/PMS, and O_3_/PMS/FeMoBC Processes for TC Degradation in Single-Factor Test

The above characterization results showed that Fe_2_Mo_3_O_8_ and FeMoO_4_ were the main components of iron molybdate composites. Therefore, in order to investigate the catalytic performance of the composite materials, the degradation efficiency of TC by O_3_, O_3_/PMS, and O_3_/PMS/FeMoBC was compared and analyzed in the experiment, and it was evaluated by 20 min degradation rate and pseudo first-order degradation kinetics (k_obs_ value).

### 4.1. Effect of Gaseous O_3_ Concentration on Degradation Efficacy

**Figure 9:**
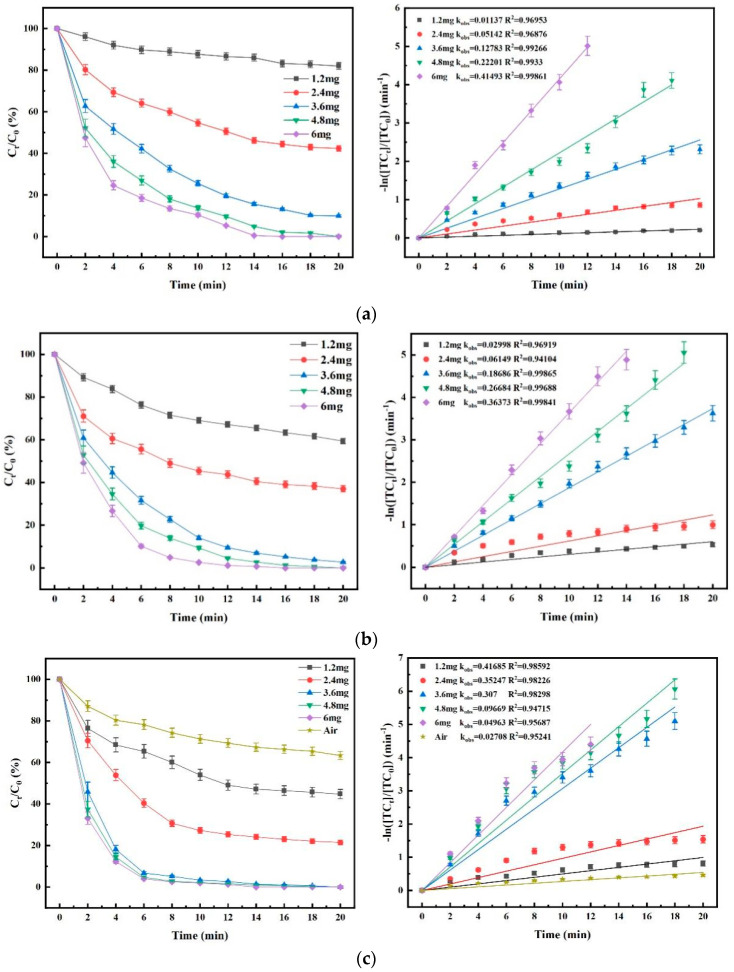
Effect of gaseous O_3_ concentration on degradation of TC and kinetic fitting. (**a**) O_3_ process; (**b**) O_3_/PMS process; (**c**) O_3_/PMS/FeMoBC process. Aqueous phase temperature: 25 ± 1 °C, FeMoBC material dosage: 200 mg/L, [TC]_0_ = 30 μM, [PMS]_0_ = 30 μM, pH = 6.8 ± 0.1, Gaseous O_3_ concentration: 1.2 mg/L, 2.4 mg/L, 3.6 mg/L, 4.8 mg/L, 6 mg/L, and Air.

As shown in Figure 9a, the degradation rate of TC decreases with the increase in O_3_ concentration in the O_3_ process within 20 min time. As shown in Figure 9b, in the O_3_/PMS process, the degradation rate of TC increases rapidly along with the degradation rate at the same time when the O_3_ concentration gradually increases, and the k_obs_ value also increases gradually. When the gaseous O_3_ concentration was 6 mg/L, the O_3_/PMS process was able to achieve a degradation rate of 92.5% in 10 min, which was a significant improvement compared with the O_3_ process and further indicated that part of the O_3_ and ^•^OH might be involved in the generation of SO_4_^•−^ at this time, thus avoiding the self-burst phenomenon caused by high ^•^OH concentration. See Equations (1)–(5) in Table 3.

In addition, the process was able to completely oxidize and remove TC from water within a 12 min reaction time period. As can be seen from Figure 9c, the effect of gaseous O_3_ concentration on the degradation efficacy in the O_3_/PMS/FeMoBC process was similar to that of the O_3_ and O_3_/PMS processes, and the k_obs_ of the reaction system increased along with the increase in the O_3_ concentration. The main reason was the shorter half-life of ^•^OH, and the self–quenching and radical competition phenomena were less frequent [4,6]. Compared with O_3_ and O_3_/PMS, the addition of FeMoBC resulted in a larger increase in the k_obs_ value, confirming the efficient catalytic efficacy of FeMoBC. In addition, the key role of O_3_ in O_3_/PMS/FeMoBC was further clarified in the Air group. Due to the low solubility in water and the fast escape rate of O_3_ from water, if the gaseous O_3_ concentration is set too high, this will result in wasted O_3_ and thus higher operating costs. Therefore, in order to reduce energy consumption and examine the coupling efficiency of O_3_ with other substances, the gaseous O_3_ concentration was set to 3.6 mg/L in this experiment while considering other influencing factors.

### 4.2. Effect of Initial TC Concentration on Degradation Efficiency

**Figure 10:**
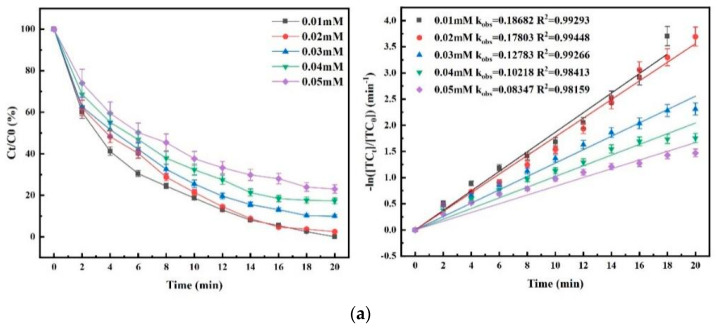

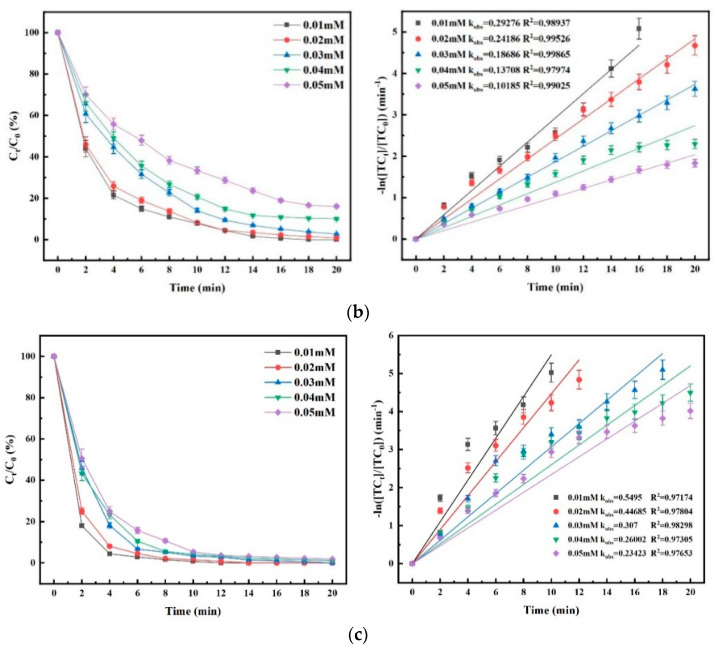
Effect of initial TC concentration on degradation rate and kinetic fitting. (**a**) O_3_ process; (**b**) O_3_/PMS process; (**c**) O_3_/PMS/FeMoBC process. Reaction conditions: aqueous phase temperature of 25 ± 1 °C, gaseous O_3_ concentration of 3.6 mg/L, FeMoBC material dosing of 200 mg/L, [PMS]_0_ = 30 μM, and solution pH of 6.8 ± 0.1. The initial TC concentrations were controlled to be 0.01 mM, 0.02 mM, 0.03 mM, 0.04 mM, and 0.05 mM.

The experiments were carried out to investigate the effects of different initial TC concentrations on the degradation efficacy of the three processes. The experimental results are shown in Figure 10.

As shown in Figure 10, the degradation rate of TC decreased with the increase in its initial concentration in the three processes. When other reaction conditions were the same, the order of TC degradation rate in the three systems from high to low was O_3_/PMS/FeMoBC > O_3_/PMS > O_3_. The main reason for the decline in the degradation rate of the three processes was that the amount of O_3_ introduced per unit time is certain, but with the increase in the concentration of target pollutants, more active oxidizing substances were needed to be consumed per unit volume, resulting in insufficient active substances, which led to the decline in the k_obs_ value. As can be seen in Figure 10c, the degradation rate of the O_3_/PMS/FeMoBC system is higher than that of both O_3_ alone and O_3_/PMS, and the reaction rate decreases with the increase in TC concentration. At a TC concentration of 0.03 mM, the k_obs_ = 0.307 min^−1^, which is 141.7% and 65.05% higher than the O_3_ and O_3_/PMS processes, respectively. Even at a TC concentration of 0.05 mM, the degradation rate of this process was still as high as 99% within 20 min.

As a result, the increase in the initial concentration of TC had a negative impact on the degradation efficacy of these three kinds of O_3_ processes. Therefore, in order to both facilitate the study and approximate the concentration in actual TC wastewater, the initial concentration of TC for subsequent studies was set to 0.03 mM.

### 4.3. Effect of PMS Concentration on Degradation Efficiency

**Figure 11:**
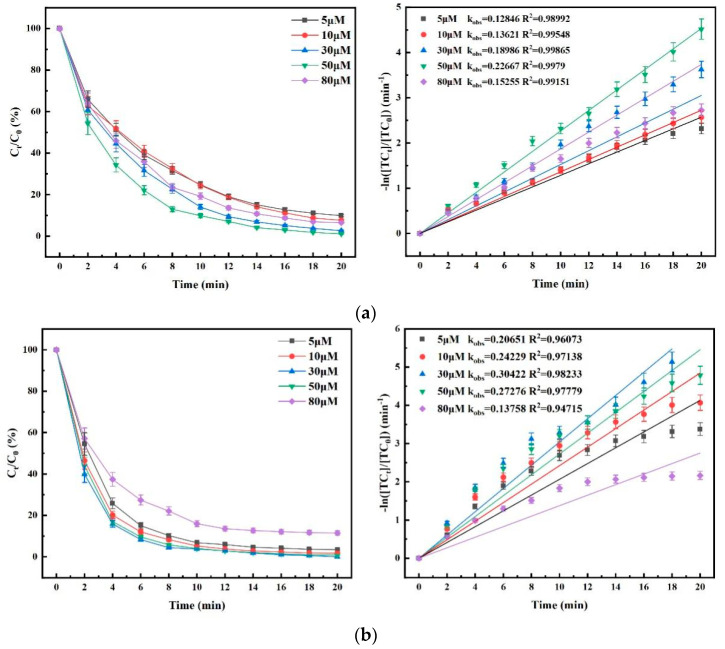
Effect of initial concentration of PMS on degradation of TC by O_3_ enhancement process and kinetic fitting. (**a**) O_3_/PMS process; (**b**) O_3_/PMS/FeMoBC process. Reaction conditions: aqueous phase temperature of 25 ± 1 °C, gaseous O_3_ concentration of 3.6 mg/L, FeMoBC material dosage of 200 mg/L, [TC]_0_ = 30 μM, and solution pH of 6.8 ± 0.1. The PMS concentration was controlled to be 5 μM, 10 μM, 30 μM, 50 μM, and 80 μM, respectively.

Figure 11 shows the effects of different PMS concentrations on the degradation efficacies of TC for the three processes (no PMS was added in the O_3_ process).

As shown in Figure 11, the influence trend of PMS concentration on the degradation efficiency of TC in O_3_/PMS and O_3_/PMS/FeMoBC systems was basically the same. That is to say, the increase in the PMS concentration had a positive effect on the improvement of TC degradation efficiency in a certain range; however, when PMS concentration increased to a critical value, the degradation rate of TC decreased. It is assumed that the main reason is that with the increase in the PMS concentration, the SO_4_^•−^ content in the reaction system increased and produced the self-quenching phenomenon [26,27]. The self-quenching formula and the reaction rate are shown in Table 4, Equations (16)–(18). This indicated that the increase in the PMS concentration was positively correlated with the catalytic efficiency of FeMoBC within a certain concentration range. However, at this time, the mutual quenching effect of free radicals that may occur when the PMS concentration was too high must be considered. Therefore, in order to fully investigate the efficacy of other single-factor experiments and to save costs, the PMS concentration was set at 30 μM in this experiment when other influencing factors were considered.

### 4.4. Effect of Material Dosage on Degradation Efficacy

**Figure 12:**
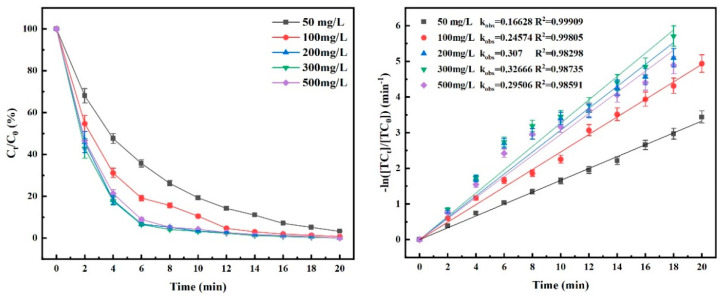
Effect of FeMoBC dosage on TC degradation by O_3_/PMS/FeMoBC and the kinetic fitting. Reaction conditions: aqueous phase temperature of 25 ± 1 °C, [PMS]_0_ = 30 μM, gaseous O_3_ concentration of 3.6 mg/L, [TC]_0_ = 0.03 mM, and solution pH of 6.8 ± 0.1; the material dosage was controlled to be 50, 100, 200, 300, and 500 mg/L.

The effect of the different dosages on TC degradation efficiency of the O_3_/PMS/FeMoBC process was investigated. The experimental results are shown in Figure 12.

As can be seen from Figure 12, the reaction rate of the O_3_/PMS/FeMoBC system was significantly enhanced compared to O_3_ and O_3_/PMS. From the figure, it can be seen that the addition of catalyst had a greater enhancement on the k_obs_ value, and the value of O_3_/PMS/FeMoBC was higher than the single O_3_ and O_3_/PMS processes under the same conditions. However, when the dosage was increased from 200 mg/L to 500 mg/L, the increase in the degradation efficiency was very small. The possible reason for this was that the rotor speed in the experimental setup was constant, and the amount of material dosed was too large, which resulted in some of the dosed material not diffusing uniformly into the reactor, leading to a slow increase in its degradation efficiency. Therefore, in order to remove the TC from the water and save costs at the same time, the material dosage was set at 200 mg/L when other influencing factors were considered in the experiment.

### 4.5. Influence of Aqueous Environment pH on TC Degradation Efficacy

**Figure 13:**
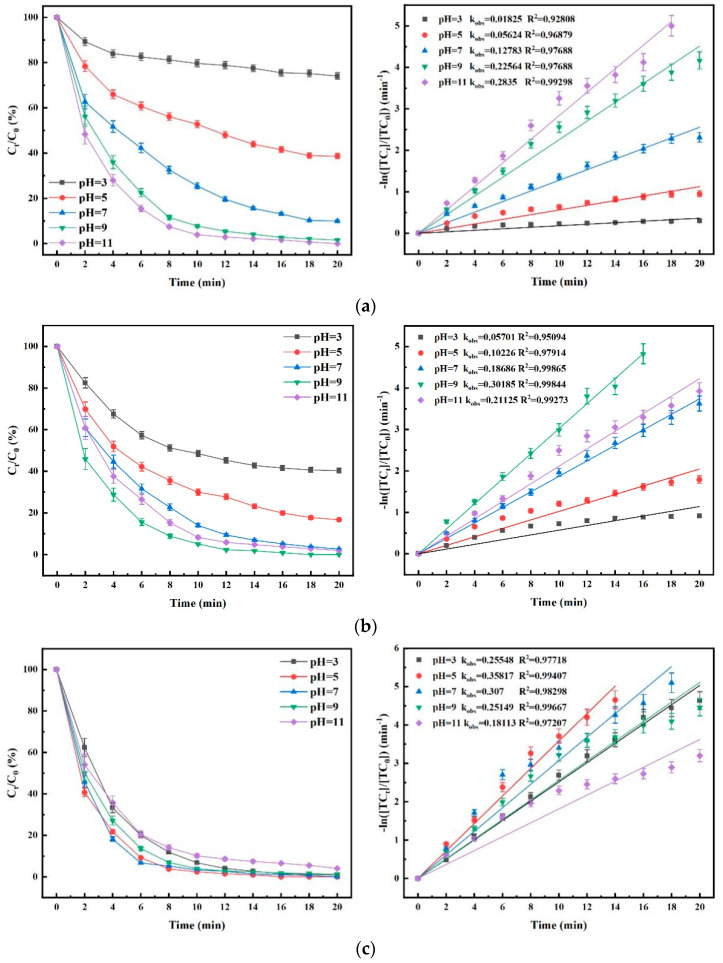
Effect of pH value of water environment on degradation of TC and kinetic fitting. (**a**) O_3_ process; (**b**) O_3_/PMS process; (**c**) O_3_/PMS/FeMoBC process. Reaction conditions: aqueous phase temperature: 25 ± 1 °C, [PMS]_0_ = 30 μM, gaseous O_3_ concentration of: mg/L, FeMoBC material dosage: 200 mg/L, [TC]_0_ = 0.03 mM, pH values of the solutions: 3.0, 5.0, 7.0, 9.0, and 11.0.

To investigate the effects of the different pH values of the water environment on the TC degradation efficacy of the three processes, the experiment was carried out, and the results are shown in Figure 13. As shown in Figure 13a, the pH value of the solution had a significant effect on the TC degradation rate. The reason for the above phenomenon is mainly due to the different oxide species reacting with TC in solutions of different pH. Under low-pH conditions, the decomposition of O_3_ is very slow due to the lack of OH^−^, which enables the accumulation of more O_3_ molecules in solution. Then the target pollutants are mainly oxidized directly by O_3_ molecules. However, when the pH of the solution increases, the concentration of OH^−^ increases accordingly. Then the OH^−^ in the system promotes the further decomposition of O_3_ molecules to generate ^•^OH which has a higher oxidizing capacity than O_3_ molecules. That is why the O_3_ process operates more efficiently under alkaline conditions.

Figure 13b shows that, in the O_3_/PMS process, the degradation of TC was optimal in weakly alkaline environments (in which the optimum was reached at pH 9.0). The literature also reported that the degradation of O_3_/PMS was optimized at pH = 9.4 [28]. The ^•^OH had the highest contribution to the degradation of TC within this system when the pH was increased from 5.0 to 7.0 but showed a decreasing trend. The degradation rate in the system gradually increased when the pH was increased from 7.0 to 9.0. This may be attributed to two phenomena: (1) the increase in hydroxyl concentration further increases the rate of O_3_ decomposition to ^•^OH, and (2) the nature of PMS-catalyzed O_3_ is the reaction with deprotonated PMS (SO_5_^2−^): $H_{2}O_{2}+2O_{3}\to {3O}_{2}+2•\mathrm{OH}$.

The secondary dissociation constant for the dissociation of PMS to SO_5_^2−^ is 9.4. Therefore, in the pH range studied, the closer the pH is to 9.4, the higher the concentration of deprotonated PMS and the faster the catalytic decomposition of O_3_. When the pH increased above 9.4, the degradation rate tended to decrease slowly. The main reason is that PMS decomposes in a non-radical manner; therefore, the rate of SO_4_^•−^ generation decreases, and too high a pH concentration causes the radicals in the system to lose their reactivity. It has also been suggested by some scholars that under strong alkaline conditions, the high concentration of OH^−^ promotes the generation of more ^•^OH, and its excess quenches SO_4_^•−^ in turn, thus affecting the alkali activation efficiency of persulfate (Equations (17) and (18)) [29,30]. The ^•^OH generated from O_3_ decomposition can react with PMS to form SO_4_^•−^, thus pH elevation promotes the generation of ^•^OH, which in turn causes an increase in SO_4_^•−^ generation [31]. In this way, the free radical content in the system was elevated, and the TC removal effect was significantly enhanced. However, when the pH was greater than 9.0, the amount of O_3_ decomposition increased, and the direct oxidation effect decreased, while the content of free radicals increased, and the mutual burst made the degradation rate of TC decrease.

As shown in Figure 13c, the degradation of TC by O_3_/PMS/FeMoBC increases and then decreases as the pH in the reaction system changes from acidic to neutral and then to weakly alkaline. The degradation rate reaches a maximum at pH = 5. This suggests that new reactive substances have been produced within the system, thereby favoring the cycling of iron ions although the acidic conditions are not conducive to the production of hydroxyl groups. Meanwhile, the better stability of O_3_ molecules will improve the efficiency of oxidative decomposition. In addition, the pH adaptation range of O_3_/PMS/FeMoBC is wider than that of the O_3_ and O_3_/PMS processes. Thus, even in the worst case of pH = 11.0 (k_obs_ = 0.18113 min^−1^), the fluctuation range is still smaller than that of the O_3_ and O_3_/PMS processes as seen from its kinetic fitting diagram. In the experiments, some of the CO_2_ in the air was dissolved in the deionized water due to the rotor stirring process. Therefore, in order to approximate the neutral pH conditions of the natural water environment, the pH settings of subsequent experiments were therefore set to 6.8 ± 0.1.

### 4.6. Effect of Common Inorganic Anions in Water on Degradation Efficacy

**Figure 14:**
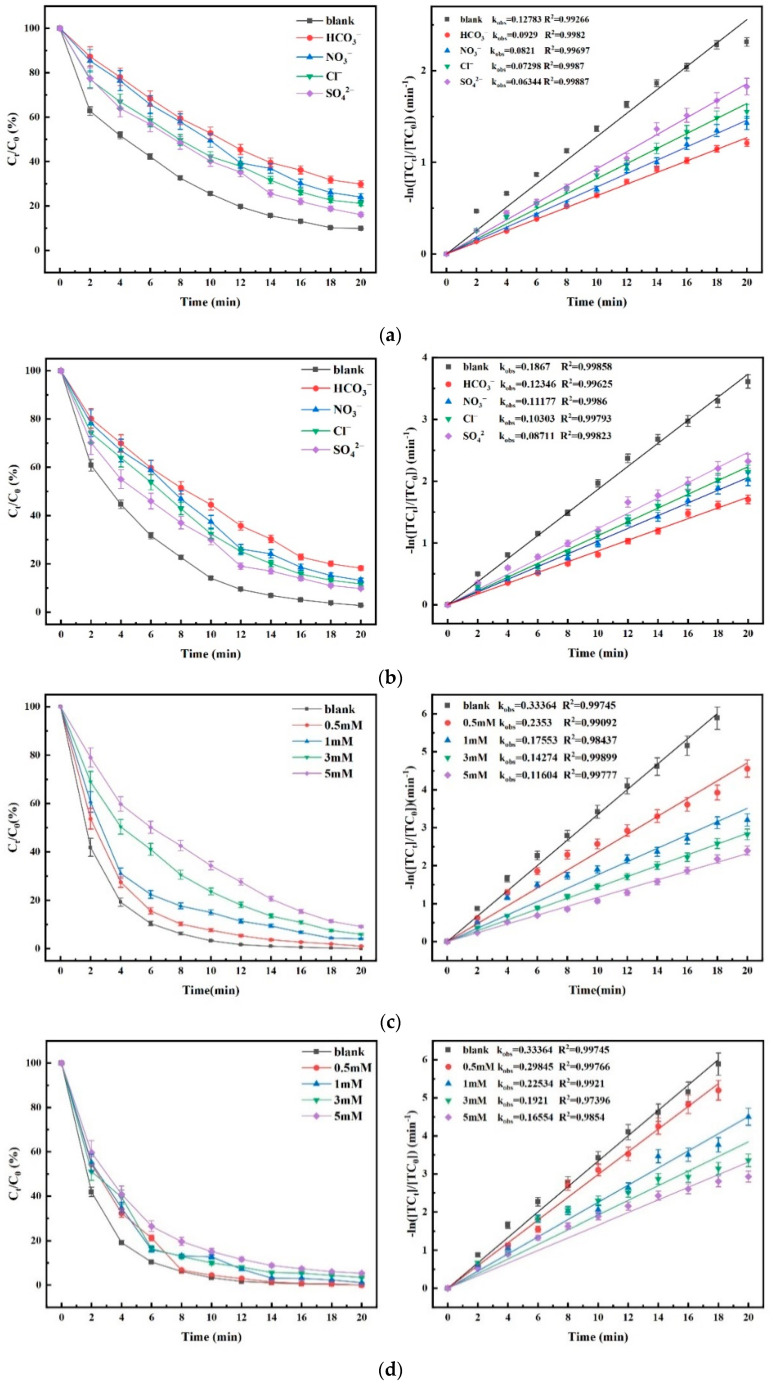

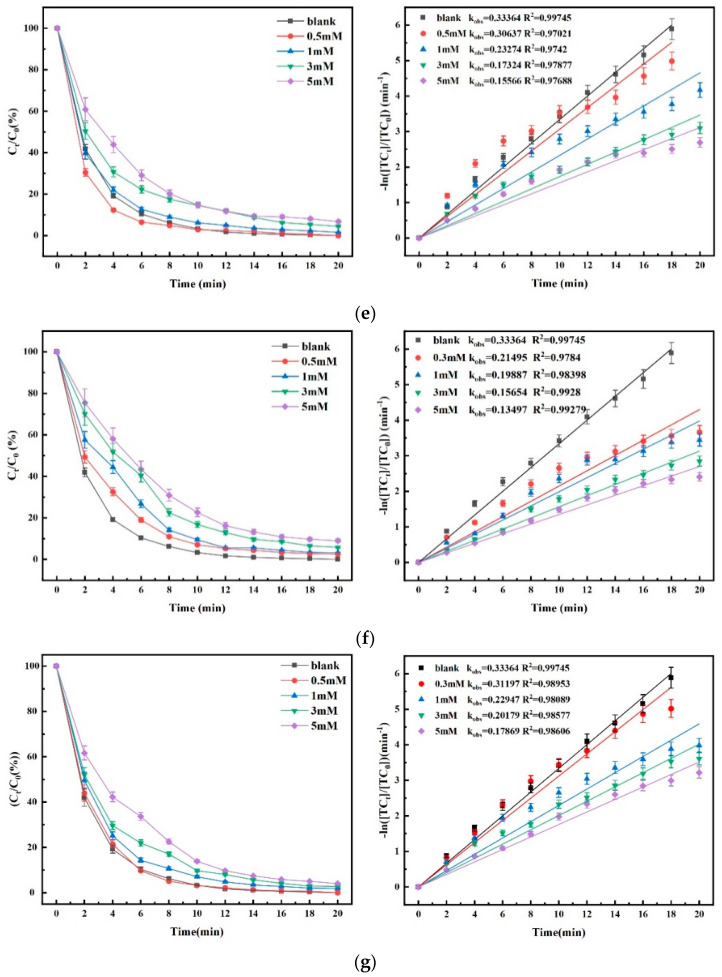
The influence of common anions on the degradation of TC and its kinetic fitting. (**a**) O_3_ process; (**b**) O_3_/PMS process; O_3_/PMS/FeMoBC process (**c**) HCO_3_^−^; (**d**) NO_3_^−^; (**e**) Cl^−^; (**f**) SO_4_^2−^; (**g**) HA. Reaction conditions: aqueous phase temperature of 25 ± 1 °C, pH of 6.8 ± 0.1, [PMS]_0_ = 30 μM, O_3_ concentration of 3.6 mg/L, [TC]_0_ = 0.03 mM, and FeMoBC material dosage of 200 mg/L. The reaction time was 20 min, and the sampling interval was 2 min.

The experiments were carried out under the control of aqueous phase temperature of 25 ± 1 °C, a pH of 6.8 ± 0.1, [PMS]_0_ = 30 μM, an O_3_ concentration of 3.6 mg/L, [TC]_0_ = 0.03 mM, and a FeMoBC material dosage of 200 mg/L. The reaction time was 20 min, and the sampling interval was 2 min. The anions are HCO_3_^−^, NO_3_^−^, Cl^−^, SO_4_^2−^, and humic acid (HA). In order to approximate the actual water anion concentration and to maximally test the anti-interference ability of the O_3_ and O_3_/PMS systems, the concentration of each anion was controlled to be 5 mM [32]. The anion concentrations of the O_3_/PMS/FeMoBC process were controlled to be 0.5 mM, 1 mM, 3 mM, and 5 mM, respectively. The blank group was under the same conditions, except that no anion was injected. The experimental results are shown in Figure 14.

As shown in Figure 14a,b, for different inorganic ions, the O_3_ and O_3_/PMS systems showed the same influence trend. Compared with the blank control group, all four inorganic ions brought some negative effects on the oxidative degradation ability of the systems, which were HCO_3_^−^, NO_3_^−^, Cl^−^, and SO_4_^2−^ in descending order. The negative effects of the other three inorganic ions decreased to different degrees, except for SO_4_^2−^, which was not significantly different from that of the single O_3_ system, indicating that the stability of the O_3_/PMS process was improved compared with that of the single O_3_ system. The active oxidants in the O_3_/PMS system were still dominated by ^•^OH, but the SO_4_^•−^ in this system enhances its anti-interference ability against various inorganic ions. For anions such as HCO_3_^−^, NO_3_^−^, and Cl^−^, which are the main interfering agents for ^•^OH and O_3_ molecules, their negative effects on the O_3/_PMS process decreased.

However, for SO_4_^2−^, the O_3_ and O_3_/PMS systems did not show significant differences, and it was speculated that the self-quenching phenomenon might have occurred after the SO_4_^•−^ concentration reached a certain level. The most obvious inhibitory effect of HCO_3_^−^ is due to the fact that HCO_3_^−^ intervenes to increase the pH of the system, and then the system changes to a weakly alkaline environment. Then the increase in OH^−^ concentration leads to the decomposition of more O_3_ molecules to produce ^•^OH. However, HCO_3_^−^ is a typical inhibitor of ^•^OH [33], so the decrease in O_3_ molecule concentration is accompanied by the depletion of ^•^OH, which reduces the overall oxidative capacity of the system [34], leading to a decrease in the TC degradation rate. The entry of NO_3_^−^ may lead to the generation of various types of NO_X_ (nitrogen oxides) in the system, which depletes part of the O_3_. Cl^−^ and SO_4_^2−^, on the other hand, may react with ^•^OH and O_3_ molecules to form the free radicals ^•^Cl, Cl_2_^•−^, and SO_4_^•−^, of which ^•^Cl and Cl_2_^•−^ have a selective oxidizing effect on N–H bonds. However, there are only two N–H bonds in the TC, so the excess ^•^Cl and Cl_2_^•−^ are likely to react with ^•^OH [35]. Meanwhile, the more stable nature of sulfate leads to the consumption of too much ^•^OH and the production of relatively little SO_4_^•−^, reducing the system’s overall oxidizing capacity.

As shown in Figure 14c–g. For the O_3_/PMS/FeMoBC system, except for chloride ion, the other common ions and HA show the trend of more and more obvious negative effects as the concentration goes from higher to lower. For Cl^−^, the k_obs_ value shows a tendency of increasing and then decreasing as the concentration increases. The principle is that when the concentration of Cl^−^ is low, the ^•^Cl produced in the system selectively breaks the N–H bond. However, when the concentration of chloride ions is increased, the substances in the aqueous phase that can be selectively oxidized by it (the N–H bond) are rapidly consumed. Then the excess ^•^Cl competes with the other reactive substances, resulting in a mutual quenching effect [36].

Table 4 shows the possible reaction formulae and reaction rates of common inorganic ions in the system. The results shows that the bursting effect of Cl^−^ on ^•^OH only works under strong acidity, so Cl^−^ only bursts SO_4_^•−^ in the reaction system under the conditions of this experiment (Equations (13) and (14) does not occur) [37,38,39,40]. NO_3_^−^ interferes with the degradation efficiency of TC by direct consumption of free radicals [41,42]. However, the inhibition degree of SO_4_^2−^ is higher than that in O_3_ and O_3_/PMS, so it is speculated that the self-quenching reaction of SO_4_^•−^ is stronger than that in the first two processes [43]. All of the above phenomena can indicate the production of new reactive substances or a greater increase in the yield of free radicals in the system.

**Table 4:** Reaction and reaction rate of common inorganic ions in O_3_/PMS/FeMoBC process.

	No.	Equation of a Chemical Reaction	Reaction Rate (M^−1^·s^−1^)	Reference
HCO_3_^−^	(6)	${\mathrm{HCO}}_{3}^{-}+•\mathrm{OH}\to {\mathrm{CO}}_{3}^{•-}+H_{2}O$	8.6 × 10^6^	[33]
	(7)	${\mathrm{HCO}}_{3}^{-}+{{\mathrm{SO}}_{4}}^{•-}\to {\mathrm{CO}}_{3}^{•-}+{{\mathrm{SO}}_{4}}^{2-}+H^{+}$	3.9 × 10^8^	[37]
NO_3_^−^	(8)	${2\mathrm{NO}}_{3}^{-}+•\mathrm{OH}\to {\mathrm{NO}}_{2}^{-}+H_{2}O$	None	[35]
	(9)	${\mathrm{NO}}_{2}^{-}+•\mathrm{OH}\to {\mathrm{NO}}_{2}^{•}+{\mathrm{OH}}^{-}$	None	[35]
	(10)	${\mathrm{NO}}_{2}^{-}+{{\mathrm{SO}}_{4}}^{•-}\to {\mathrm{NO}}_{2}^{•}+{{\mathrm{SO}}_{4}}^{2-}$	None	[35]
	(11)	${\mathrm{NO}}_{2}^{-}+{\mathrm{HSO}}_{5}^{-}\to {\mathrm{NO}}_{3}^{-}+{\mathrm{HSO}}_{4}^{-}$	None	[36]
	(12)	${\mathrm{NO}}_{2}^{-}+O_{3}\to {\mathrm{NO}}_{3}^{-}+O_{2}$	None	[36]
Cl^−^	(13)	$•\mathrm{OH}+{\mathrm{Cl}}^{-}\to {\mathrm{ClOH}}^{•-}$	4.3 × 10^9^	[38]
	(14)	${\mathrm{ClOH}}^{•-}\to •\mathrm{OH}+{\mathrm{Cl}}^{-}$	6.1 × 10^9^	[38]
	(15)	${{\mathrm{SO}}_{4}}^{•-}+{\mathrm{Cl}}^{-}\to {{\mathrm{SO}}_{4}}^{2-}+{\mathrm{Cl}}^{•}$	3.0 × 10^8^	[39]
	(16)	${{\mathrm{SO}}_{4}}^{2-}+{\mathrm{Cl}}^{•}\to {{\mathrm{SO}}_{4}}^{•-}+{\mathrm{Cl}}^{-}$	2.5 × 10^8^	[39]
	(17)	${\mathrm{Cl}}^{-}+{\mathrm{Cl}}^{•}\to {\mathrm{Cl}}_{2}^{•-}$	8.5 × 10^9^	[39]
	(18)	${\mathrm{Cl}}_{2}^{•-}\to {\mathrm{Cl}}^{-}+{\mathrm{Cl}}^{•}$	6.0 × 10^4^	[40]
	(19)	${\mathrm{ClOH}}^{•-}+H^{+}\to {\mathrm{Cl}}^{•}+H_{2}O$	2.1 × 10^10^	[37]
	(20)	${\mathrm{Cl}}_{2}^{•-}+•\mathrm{OH}\to \mathrm{HOCl}+{\mathrm{Cl}}^{-}$	1.0 × 10^9^	[40]
SO_4_^2−^	(21)	${{\mathrm{SO}}_{4}}^{•-}+{{\mathrm{SO}}_{4}}^{•-}\to S_{2}O_{8}^{2-}$	None	[39]
	(22)	${{\mathrm{SO}}_{4}}^{•-}+•\mathrm{OH}\to HSO_{4}^{•-}+1/2O_{2}$	None	[39]
	(23)	${{\mathrm{SO}}_{4}}^{•-}+S_{2}O_{8}^{2-}\to SO_{4}^{2-}+S_{2}O_{8}^{-}$	None	[39]

## 5. O_3_/PMS/FeMoBC Reaction Mechanism Speculation

The above analysis on the degradation efficiency of the O_3_/PMS/FeMoBC system for TC was similar to the existing experimental studies. For example, the research showed that 95% of orange G could be removed by the FeMoO_4_/PS system after 40 min. ^•^OH and SO_4_^•−^ existed in the system at the same time, and SO_4_^•−^ was the main active substance in the oxidation process [44]. In addition, Fe_2_(MoO_4_)_3_ also maintained good catalytic performance for activating persulfate in Fenton reaction system [45,46]. Although ^•^OH and SO_4_^•−^ have been proved to be the main reactive oxygen species (ROS) in iron-based molybdate/PS system, recent reports also showed the potential of coexistence of non-radical mechanisms in an iron-based activated PMS or PS system. For instance, Zhang et al. reported that singlet oxygen (^1^O_2_) played a key role in the removal of acetaminophen in the Fe^2+^/MoS_2_/PMS system [47]. Yang et al. reported that in an Mn-Fe carbon oxide/PMS system, ^1^O_2_ could be used as a secondary driving force to promote the generation of ^•^OH and SO_4_^•−^, thus accelerating the oxidation process [48]. Although iron molybdate has been reported in the literature, the specific catalytic activation mechanism remains to be discussed, especially the contribution of non-free radicals in an iron-based molybdate/PMS system is not clear. Therefore, the degradation mechanism of TC in O_3_/PMS/FeMoBC system was speculated by EPR technology and a quenching experiment.

### 5.1. Free Radical Burst Experiments for O_3_, O_3_/PMS, and O_3_/PMS/FeMoBC

In this study, the types and contribution of active oxidants of these three processes were explored by free radical burst experiments. Tert–butanol (TBA), ethanol (EtOH), L–histidine, and p–benzoquinone (p–BQ) were used for ^•^OH, SO_4_^•−^, single linear oxygen (^1^O_2_), and superoxide radicals (•O_2_^−^), respectively. Among them, the reaction rate constants of TBA with ^•^OH were 3.8–7.6 × 10^8^ M^−1^s^−1^ for quenching, and those of EtOH with ^•^OH and SO_4_^•−^ were 1.2–2.8 × 10^9^ M^−1^s^−1^ and 1.6~7.7 × 10^7^ M^−1^s^−1^, with high k_obs_ values for both ^•^OH and SO_4_^•−^, which can quench both radicals simultaneously; L–histidine has a reaction rate constant of 3.2 × 10^8^ M^−1^s^−1^ with ^1^O_2_ [49], and p–BQ has a reaction rate constant of 1.1 × 10^9^ M^−1^s^−1^ with •O_2_^−^ [50], which can be used as quenchers of ^1^O_2_ and •O_2_^−^, respectively.

For the O_3_/PMS process, on the basis of the quenching of ^•^OH by TBA, the quenching experiment of SO_4_^•−^ by EtOH was added. While for the O_3_/PMS/FeMoBC process, according to the experimental phenomenon above, it may produce other active substances; so on the basis of using TBA and EtOH, L–histidine and p–BQ were added to identify the presence of ^1^O_2_ and •O_2_^−^, respectively.

**Figure 15:**
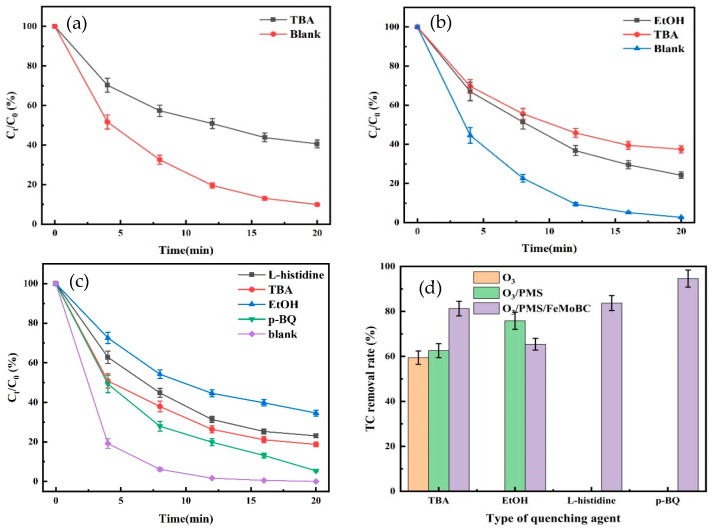
O_3_, O_3_/PMS, and O_3_/PMS/FeMoBC quenching experiment. (**a**) O_3_ quenching experiment; (**b**) O_3_/PMS quenching experiment; (**c**) O_3_/PMS/FeMoBC quenching experiment; (**d**) influence of each system on the degradation of TC under the quenching agent.

The reaction conditions for the quenching experiments were as follows: reaction temperature of 25 ± 1 °C, gaseous O_3_ concentration of 3.6 mg/L, initial concentration of TC of 0.03 mM, solution pH of 6.8 ± 0.1, PMS concentration of 30 μM for the O_3_/PMS and O_3_/PMS/FeMoBC processes, and a dosage of FeMoBC material of 200 mg/L for the O_3_/PMS/FeMoBC process. The dosage of all quenching agents was 100 mM. The quenching experiments of O_3_, O_3_/PMS, and O_3_/PMS/FeMoBC processes on various types of radicals were investigated under the above conditions. The experimental results are shown in Figure 15.

Figure 15a shows that the degradation rate of TC by O_3_ process decreased to 59.4% within 20 min with the input of excess TBA, indicating that ^•^OH does not play a major role in the single O_3_ process, but it is the O_3_ molecules that play a major role in oxidation. Figure 15b shows that the degradation rate of TC by O_3_/PMS decreased from 97.3% to 62.5% and 75.8%, respectively, after the addition of TBA and EtOH. The rate constants for the reaction of TBA with ^•^OH and SO_4_^•−^ were 3.8–7.6 × 10^8^ M^−1^s^−1^ and 4.0~9.1 × 10^5^ M^−1^s^−1^, respectively, and ^•^OH was preferentially burst in the presence of ^•^OH. At this time, part of the ^•^OH in the system was used to convert SO_4_^•−^, so the effect of TBA on the ^•^OH was lower than that of single O_3_. However, EtOH had a quenching effect on both ^•^OH and SO_4_^•−^, so it had a greater effect on the degradation efficiency of the O_3_/PMS process.

For the O_3_/PMS/FeMoBC process, the experiments result above showed that it may produce other active substances, so the L–histidine and p–BQ were added to the experiment to identify the possible ^1^O_2_ and •O_2_^−^, respectively. As shown in Figure 15c, the degradation of TC by the O_3_/PMS/FeMoBC process decreased from 100% to 81.3%, 65.4%, 76.9%, and 94.6%, respectively, after the addition of TBA, EtOH, L–histidine, and p–BQ. Among them, EtOH brought the most negative effect to the O_3_/PMS/FeMoBC process, indicating that the main active substances in the reaction system were still ^•^OH and SO_4_^•−^. Meanwhile, L–histidine and p–BQ decreased the TC degradation efficiency by 23.1% and 5.4%, respectively, suggesting that single linear oxygen (^1^O_2_) and superoxide radicals (•O_2_^−^) may have been generated in the O_3_/PMS/FeMoBC process. To further investigate the presence of ^1^O_2_ and •O_2_^−^ in the system, it is necessary to identify them by EPR detection. Figure 15d shows the TC removal rates of the O_3_, O_3_/PMS, and O_3_/PMS/FeMoBC systems in the presence of each quencher background. In the TBA item, the O_3_ process was the least affected, followed by O_3_/PMS, and O_3_/PMS/FeMoBC was the most affected, which confirms that O_3_/PMS/FeMoBC has a stronger ^•^OH yield. The same trend was shown in the EtOH quenching test, and the quenching experiments of L–histidine and p–BQ indicated the possible presence of ^1^O_2_ and •O_2_^−^ in the O_3_/PMS/FeMoBC process.

### 5.2. Analysis of Free Radical Species for TC Degradation by O_3_/PMS/FeMoBC

**Figure 16:**
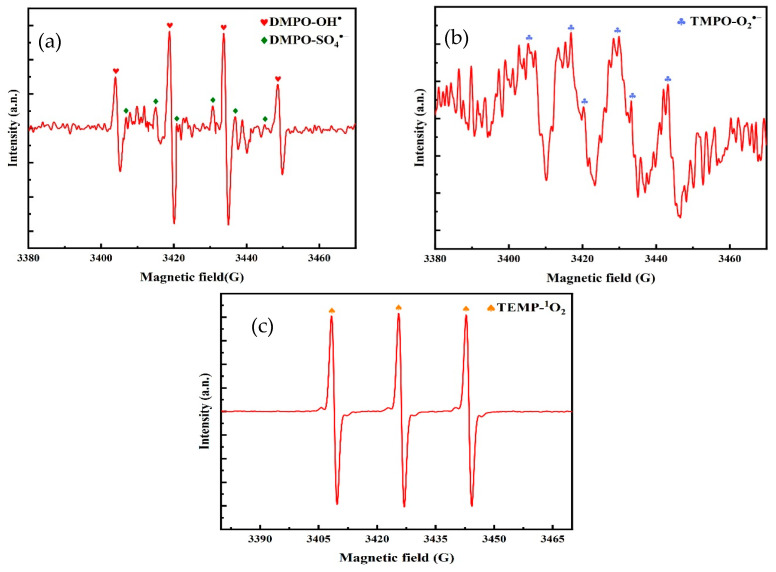
EPR map of active species in O_3_/PMS/FeMoBC system. (**a**) DMPO–^•^OH, DMPO–SO_4_^•−^; (**b**) DMPO–•O_2_^−^; (**c**) TEMP–^1^O_2_.

In order to verify the generation of reactive species in the O_3_/PMS/FeMoBC system, 5,5–dimethyl–1–pyrroline–N–oxide (DMPO) and 2,2,6,6–tetramethylpiperidinium–N–oxide (TEMPO) were employed as spin trapping agents in this study, and these reactive species were characterized by the electron paramagnetic resonance (EPR) technique. DMPO, as a multifunctional trapping agent, was able to react with ^•^OH, SO_4_^•−^, and •O_2_^−^ to form characteristic DMPO–^•^OH, DMPO–SO_4_^•−^, and DMPO–•O_2_^−^ adducts, respectively. TEMPO, on the other hand, is specifically designed for the detection of single–linear state oxygen, with which it reacts to form TEMPO–^1^O_2_ adducts. In this study, DMPO–•OH and DMPO–SO_4_^•−^ adducts in the ratio of 1:2:2:1 and 1:1:1:1:1:1:1:1 were observed in the O_3_/PMS/FeMoBC system by EPR analysis (shown in Figure 16), and this result indicate that the system indeed produces both ^•^OH and SO_4_^•−^ radicals. At the same time, a weak DMPO–•O_2_^−^ signal (1:1:1:1:1 ratio) and a 1:1:1 ratio TEMPO–^1^O_2_ adduct signal were captured. The above conclusions indicate that not only the three free radicals ^•^OH, SO_4_^•−^, and •O_2_ but also the non–radical form of ^1^O_2_ exists in the O_3_/PMS/FeMoBC system. This result is in agreement with the data from the free radical burst experiments in the previous section.

**Figure 17:**
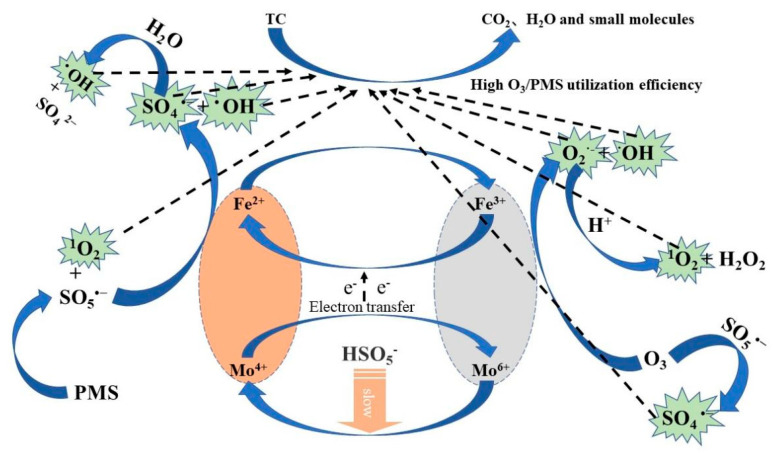
O_3_/PMS/FeMoBC system reaction flowchart.

Based on the quenching experiments and EPR radical identification, the reaction mechanism of the O_3_/PMS/FeMoBC process can be deduced. That is, in the O_3_/PMS/FeMoBC reaction system, O_3_ can accept electrons from Fe^2+^ and in this way generates ^•^OH radicals and •O_2_^−^ radicals (Equations (24)~(26)) [51]. Ferrous ions in solution subsequently react with –•O_2_^−^ to form H_2_O_2_, which reacts with Fe^2+^ in a Fenton-like reaction to produce more hydroxyl radicals (Equations (27)~(29)) [52]. PMS hydrolyses to HSO_5_^−^ and SO_5_^−^ and further produces ^1^O_2_ (Equation (30)). Accompanying the gradual progress of the reaction, Mo^4+^ exposed on the catalyst surface reacts with Fe^3+^ (Equation (31)), accelerating the regeneration of Fe^2+^, which in turn undergoes a series of redox reactions with PMS to generate free radicals (Equation (32)) [46]. Meanwhile, HSO_5_^−^ produced by PMS hydrolysis can undergo reduction reactions with a portion of Fe^3+^ and Mo^6+^ (Equations (33) and (34)) [51]. Hydrogen ions in solution can lead to the protonation of Mo^6+^ to produce Mo (VI) peroxo–complexes MoO (OH)(O_2_)_2_^−^ and further production of singlet oxygen (Equations (35) and (36)) [53]. The reactions that may be involved in the O_3_/PMS system for FeMoBC are shown in Table 5, and the reaction flow diagram is shown in Figure 17.

For bimetallic catalysts dominated by elemental iron, the electronic and geometrical structures differ from those of the corresponding monometallic catalysts. However, the correlation between reactivity and structural factors is still unknown, and some researchers believe [45] that another metallic element acts as an electron donor, and the main reaction is realized by the coupling of iron to the oxidant (Fenton and Fenton-like). However, the doping of the element may bring about new electronic structures and geometrical changes (e.g., oxygen vacancies). In most reactions, bimetallic catalysts exhibit enhanced catalytic performance (higher activity or selectivity) [55]. However, the reason why bimetallic catalysts are able to provide better or different performance than the corresponding monometallic catalysts is still unclear.

## 6. Conclusions

In this study, compared with O_3_ and O_3_/PMS processes, the efficiency, degradation kinetics, and mechanism of O_3_/PMS/FeMoBC process for removing TC from water were analyzed. The following conclusions are drawn:

(1) SEM–EDS, FTIR, BET, XRD, XPS, and other analysis results showed that Mo and Fe were stably loaded on the surface and pores of BC during the preparation of iron molybdate composite, forming more oxygen-containing functional groups of Mo and Fe. The main components of the composite catalytic material were Fe_2_Mo_3_O_8_ and FeMoO_4_, accounting for 26.3% and 73.7%, respectively. In addition, the catalytic reaction was mainly concentrated on the catalyst surface.
(2) O_3_, O_3_/PMS, and O_3_/PMS/FeMoBC processes all could effectively remove TC from water, among which the O_3_/PMS/FeMoBC process had the best effect. The results of single factor experiments show that in the O_3_/PMS/FeMoBC process, the degradation rate of TC decreased with the increase in its initial concentration and increased with the increase in O_3_ concentration. The best pH condition is weak acidity. Too high of an oxidant concentration would reduce the degradation effect of TC, which was consistent with the trend of the other two methods. However, the O_3_/PMS/FeMoBC system showed stronger anti-interference ability.
(3) Quenching experiments were carried out using TBA, EtOH, L–histidine, and p–BQ to quench the free radicals generated in the O_3_, O_3_/PMS, and O_3_/PMS/FeMoBC systems, respectively. The results showed that the O_3_ molecule played the main oxidizing role in the O_3_ system alone, and both ^•^OH and SO_4_^•−^ reactive substances were present in the O_3_/PMS system. For the O_3_/PMS/FeMoBC system, EtOH had the greatest effect on it, suggesting that ^•^OH and SO_4_^•−^ were the main reactive substances in the system. L–histidine and p–BQ decreased the degradation efficiency during the chemical actions, suggesting that ^1^O_2_ and •O_2_^−^ may be generated in this system. Combined with the above free radical quenching experiments and the analysis results of EPR technique, it could be concluded that four active oxidants, ^•^OH, SO_4_^•−^, ^1^O_2_, and •O_2_^−^, were present in the O_3_/PMS/FeMoBC system, and their contributions were, in descending order, ^•^OH, ^1^O_2_, SO_4_^•−^, and •O_2_^−^.

## Figures and Tables

**Table 1:** Experimental reagents in the experiment.

Reagent	Chemical Formula	Pureness	Manufacturer
Tetracycline hydrochloride	C_22_H_24_N_2_O_8_•HCl	≥99%	Shanghai McLean Biochemical Technology Co. (Shanghai, China)
Sodium hyposulfide	Na_2_S_2_O_3_	AR	Sinopharm Chemical Reagent Co. (Shanghai, China)
Ferric chloride hexahydrate	Fecl_3_•6H_2_O_2_	AR	Sinopharm Chemical Reagent Co. (Shanghai, China)
Sodium chloride	NaCl	AR	Sinopharm Chemical Reagent Co. (Shanghai, China)
Methanoic acid	HCOOH	≥99%	Shanghai McLean Biochemical Technology Co. (Shanghai, China)
Sodium nitrate	NaNO_3_	AR	Sinopharm Chemical Reagent Co. (Shanghai, China)
Tertiary butyl alcohol	C_4_H_10_O	AR	Sinopharm Chemical Reagent Co. (Shanghai, China)
Sodium bicarbonate	NaHCO_3_	AR	Sinopharm Chemical Reagent Co. (Shanghai, China)
L–Histidine	C_6_H_9_N_3_O_2_	≥99%	Shanghai McLean Biochemical Technology Co. (Shanghai, China)
Potassium peroxymonosulfate	KHSO_5_	AR	Sigma–Aldrich Ltd. (St. Louis, MO, USA)
Methyl alcohol	CH_3_OH	Superior Pure	Sigma–Aldrich Ltd. (St. Louis, MO, USA)
Sodium molybdate	Na_2_MoO_4_	AR	Sinopharm Chemical Reagent Co. (Shanghai, China)
P–benzoquinone	C_6_H_4_O_2_	≥99%	Shanghai McLean Biochemical Technology Co. (Shanghai, China)

**Table 2:** Pore properties of BC, FeBC, MoBC, FeMoBC (new), and FeMoBC (used).

Sample	S_BET_ (m^2^g^−1^)	S_micro_ (m^2^g^−1^)	V_total pore_ (cm^3^g^−1^)	D_aver_ (nm)
BC	28.8771	18.0232	0.031685	4.3890
FeBC	151.4387	110.0570	0.091465	2.4159
MoBC	160.4957	94.4786	0.139421	3.4748
FeMoBC (new)	80.3445	44.5264	0.094498	4.7046
FeMoBC (used)	82.7599	58.6966	0.102354	4.9470

**Table 3:** Equations of ^•^OH formation promoted by PMS and SO_4_^•−^.

No.	Equation of a Chemical Reaction	References
(1)	${{\mathrm{HSO}}_{5}}^{-}+H_{2}O\to H{{\mathrm{SO}}_{4}}^{-}+H_{2}O_{2}$	[23]
(2)	${{\mathrm{SO}}_{5}}^{2-}+H_{2}O\to {{\mathrm{SO}}_{4}}^{2-}+H_{2}O_{2}$	[24]
(3)	$H_{2}O_{2}+2O_{3}\to {3O}_{2}+2•OH$	[24]
(4)	${{\mathrm{SO}}_{4}}^{•-}+OH^{-}\to {{\mathrm{SO}}_{4}}^{2-}+•\mathrm{OH}$	[25,26]
(5)	${{\mathrm{SO}}_{4}}^{•-}+H_{2}O\to H^{+}+{{\mathrm{SO}}_{4}}^{2-}+•OH$	[27]

**Table 5:** Possible reactions involved in the O_3_/PMS/FeMoBC system.

No.	Equation of a Chemical Reaction	Reference
(24)	${Fe}^{2+}+O_{3}+H^{+}\to {Fe}^{3+}+•OH+O_{2}$	[51]
(25)	${Fe}^{2+}+O_{3}\to {FeO}^{2+}+O_{2}$	[51]
(26)	${Fe}^{2+}+O_{3}\to {Fe}^{3+}+{O_{2}}^{•-}$	[51]
(27)	${Fe}^{2+}+{O_{2}}^{•-}+{2H}^{+}\to {Fe}^{3+}{+H}_{2}O_{2}$	[52]
(28)	${Fe}^{2+}{+H}_{2}O_{2}\to {Fe}^{3+}+•OH+OH^{-}$	[52]
(29)	${FeO}^{2+}+H_{2}O\to •OH+{Fe}^{3+}+OH^{-}$	[52]
(30)	HSO5−+SO52−+OH−→O21+SO52−+H2O	[46]
(31)	${Mo}^{4+}+{Fe}^{3+}\to {Mo}^{6+}+{Fe}^{2+}$	[53]
(32)	${Fe}^{2+}+{{\mathrm{HSO}}_{5}}^{-}\to {Fe}^{3+}+{{\mathrm{SO}}_{4}}^{•-}+OH^{-}$	[46]
(33)	${Fe}^{3+}+{{\mathrm{HSO}}_{5}}^{-}\to {Fe}^{2+}+{{\mathrm{SO}}_{5}}^{•-}+H^{+}$	[51]
(34)	${Mo}^{6+}+{{\mathrm{HSO}}_{5}}^{-}\to {Mo}^{4+}+{{\mathrm{SO}}_{5}}^{•-}+H^{+}$	[51]
(35)	$MoO_{3}+H^{+}\to {HMoO_{3}}^{+}$	[54]
(36)	${HMoO_{3}}^{+}+{{\mathrm{HSO}}_{5}}^{-}\to {Mo{O(OH)(O_{2})}_{2}}^{-}{{+\mathrm{SO}}_{4}}^{2-}+H_{2}O$	[54]
(37)	MoO(OH)(O2)2−+H2O→MoO42−+O21+H+	[54]

## Data Availability

Data are contained within the article.
